# Advances in studies of disease-navigating webs: *Sarcoptes scabiei* as a case study

**DOI:** 10.1186/1756-3305-7-16

**Published:** 2014-01-09

**Authors:** Samer Alasaad, Mathieu Sarasa, Jorg Heukelbach, Domnic Mijele, Ramón C Soriguer, Xing-Quan Zhu, Luca Rossi

**Affiliations:** 1 Estación Biológica de Doñana, Consejo Superior de Investigaciones Científicas (CSIC), Avda. Américo Vespucio s/n 41092, Sevilla, Spain; 2 Institute of Evolutionary Biology and Environmental Studies (IEU), University of Zürich, Winterthurerstrasse 190, 8057 Zürich, Switzerland; 3 Grupo Biología de las Especies Cinegéticas y Plagas (RNM—118), Sevilla, Spain; 4 Fédération Nationale des Chasseurs, 13 rue du Général Leclerc, 92136 Issy les Moulineaux, France; 5 Department of Community Health, School of Medicine, Federal University of Ceará, Fortaleza, Brazil; 6 Kenya Wildlife Service, P.O. Box 40241-00100, Nairobi, Kenya; 7 State Key Laboratory of Veterinary Etiological Biology, Key Laboratory of Veterinary Parasitology of Gansu Province, Lanzhou Veterinary Research Institute, Chinese Academy of Agricultural Sciences, Lanzhou, Gansu Province 730046, P R China; 8 Dipartimento di Scienze Veterinarie, Università degli Studi di Torino, Via Leonardo da Vinci 44, I-10095 Grugliasco, Italy

**Keywords:** Epidemiology, Disease, Genetics, Ecology, Navigating-web, *Sarcoptes* mite, Integrated approach

## Abstract

The discipline of epidemiology is the study of the patterns, causes and effects of health and disease conditions in defined *anima* populations. It is the key to evidence-based medicine, which is one of the cornerstones of public health. One of the important facets of epidemiology is disease-navigating webs (disease-NW) through which zoonotic and multi-host parasites in general move from one host to another. Epidemiology in this context includes (i) classical epidemiological approaches based on the statistical analysis of disease prevalence and distribution and, more recently, (ii) genetic approaches with approximations of disease-agent population genetics. Both approaches, classical epidemiology and population genetics, are useful for studying disease-NW. However, both have strengths and weaknesses when applied separately, which, unfortunately, is too often current practice. In this paper, we use *Sarcoptes scabiei* mite epidemiology as a case study to show how important an integrated approach can be in understanding disease-NW and subsequent disease control.

## Review

### Introduction

Epidemiology is the study of the patterns, causes and effects of health and disease conditions in defined *anima* populations. One of the important facets of epidemiology is disease-navigating webs (disease-NW), through which parasites move from one host to another [1-4]. Classical epidemiology is useful in cases of population-based and ecological studies that rely on statistical analyses of disease prevalences and distributions. However, in recent years increasingly more studies have been conducted using approximations of disease-agent population genetics. Both approaches, that is, classical epidemiological studies and genetics, are useful for studying disease-NW. However, both have strengths and weaknesses when they are applied separately, which is unfortunately current practice in many cases due to a number of reasons:

– A temporal gap in the research, usually in classical field studies, that affects pathological agent collection and conservation, but also more recent genetics-based studies of the pathological agent.

– The nature of genetic studies, namely, the small size of the pathological agent, which hampers disease-agent collection and good-quality DNA extraction. This led some disease-NW research to rely on classical-type studies, which did not necessarily require the isolation of the pathological agent since they can be based on clinical or histopathological examinations or on indirect diagnostic methods. Fortunately, the new DNA extraction and PCR kits allow DNA extraction and amplification from tiny amounts of almost every biological material.

– The relatively high cost of the genetics-based research and the need for appropriate infrastructure. This is a burden for this type of research, especially in developing countries and in laboratories that suffer from highly variable or unpredictable financial support.

– The formation of different specialized research groups has led to research that relies on either population-based or genetics-based studies, which further highlights the need for collaboration in research, as proposed by the *Sarcoptes*-World Molecular Network (*Sarcoptes*-WMN) [5]. These collaborations include the exchange of information and infrastructure, especially among researchers in the same region. If these key points are addressed, these specialized groups become more active and research will be directed to a common goal, which is the eradication of the disease and understanding its dynamics.

### Classical epidemiological studies

In classical population-based and ecological studies, the description of disease-navigating webs is based on the analysis of differences in the temporal and spatial distribution of disease prevalence between different host species, sexes, ages and geographical locations [6-10]. This approach is often included within the ecological exploration of the potential causes and effects of parasite transmission [11-15]. Such studies are relatively cheaper than genetic approaches and numerous statistical software packages are available for robust analyses (e.g. free software environments for statistical computing and graphics such as the R Development Core Team [16]). The main disadvantages of classical epidemiological studies include (a) the lack of robustness in analyses due to low sample size, the overabundance of healthy individuals in data sets and missing data; fortunately recent statistical approaches have been developed that can overcome these handicaps [17,18]; (b) in some cases we misunderstand or assume we know the directionality of disease navigation, and we limit our understanding to the correlation between just two host species, sexes, ages or localities [19,20], which can be misleading, above all when the epidemiological study is carried out *a posteriori* and is not monitored during the progress of a disease outbreak. Hence there is a need of the immediate and real-time monitoring of disease outbreaks; (c) disease outbreaks in many cases evolve very rapidly, well beyond the capabilities of monitoring and sampling programmes, which limits the potential of the available statistical tools to fully describe disease-navigating webs [21,22]; (d) raw data are usually not published and the reuse and analysis of data by other research groups requires direct collaboration between laboratories and research networks [5], Scientists should find a way to publish the raw data as supplementary material together with their publications.

### Genetics-based studies

The genetic description of disease-NW is based on the mitochondrial or chromosomal nucleotide differences between disease-agent populations or the allelic frequency of different genetic loci such as microsatellites and single-nucleotide polymorphisms (SNPs) [23-31]. Such genetics-based studies have certain advantages over the classical epidemiological studies that include the fact that (a) they are usually robust even with relatively low sample size [24,25]; (b) they permit studies of a wide-ranging disease-navigating web among individuals of the same host species, between different host species and even within the same individual animal [25,32,33]; (c) these tools are especially useful when the large spatial-temporal distribution of a disease would make spatial-temporal monitoring using classical methods far more complex [32]; (d) they are useful for *a posteriori* epidemiological studies since molecular markers are usually stable over time [27], which allows the monitoring of the navigating web over extended time intervals; (e) they enable raw data from other research groups to be reused, since sequences are usually uploaded onto appropriate databases [26]; nevertheless, this is not the case of the allelic frequency of different genetic loci such as microsatellites and SNPs [28] because to date there is no specialized site for uploading such data; and (f) they are usually performed within a relatively short period of time.

On the other hand, genetics-based studies are usually hampered by (a) the difficulties involved in the collection and conservation of pathological agents and in the extraction of good-quality DNA [34,35]; (b) the need for adequate molecular markers, since in phylogenetic analyses bootstrapping support for the closest relationships may be relatively poor if not enough time has elapsed for informative changes in the sequences examined to have accumulated. However, further resolution can be achieved using faster evolving hypervariable sequences such as nuclear polymorphic microsatellite loci and SNPs, and hence the need of developing new genetic markers for the unstudied organisms, which can be in some cases time-consuming, labour-intensive and expensive [30]; (c) the fact that genetic data does not necessarily reveal the ecological causal and concomitant factors for disease navigation; and (d) the fact that genetics-based studies are still relatively expensive and require good infrastructure.

### *Sarcoptes scabiei* as a case study

The neglected parasitic mite *Sarcoptes scabiei* affects humans and a wide range of mammalian hosts worldwide [36,37]. Scabies (or mange as it called in animals) is an emerging/re-emerging disease.

Relatively little genetics-based research on *S. scabiei* has been conducted in comparison with classical epidemiological research. This is primarily due to difficulties in obtaining suitable skin samples from hosts, in isolating sufficient numbers of mites and in extracting adequate amounts of genetic material from individual mites [19,34,35,38]. Due to certain inefficiencies at several points, a variable number of failed preparations have been reported [29]. Classical epidemiological studies do not need the regular isolation of *Sarcoptes* mites and can be carried out using clinical, gross pathological and histopathological examinations or indirect diagnostic methods [39].

Genetics-based studies of *Sarcoptes* mites have also been affected by the difficulty in selecting genetic markers. Using the ITS-2 sequences, Zahler *et al.*[23] and Alasaad *et al*. [26] failed to find any clear-cut evidence of genetic separation between mite populations, a finding that could be related to the host species in question or geographical location. The phylogenetic analysis bootstrapping support for the closest relationships may be relatively poor if not enough time has elapsed for informative changes in the sequences examined to have occurred. Conversely, the use of microsatellite markers has been appropriate in the study of *Sarcoptes*-NW [5]. Obviously, ecological and classical population-based studies do not suffer from this disadvantage.

Genetics-based studies of *Sarcoptes*-NW are also affected by the choice of the analytic model and the correct interpretation of the results. Skerratt *et al.*[40] phylogenetically analysed sequence data for several haplotypes of *S. scabiei* from humans, dogs and wombats in Australia to test scenarios concerning the origin and diversification of scabies infection in wombats. Their results suggest that the *Sarcoptes* mite was only relatively recently introduced into Australia by colonizers and/or their dogs. However, these conclusions have been called into question due to the choice of the model used in the phylogenetic analysis, the lack of a root for the phylogenetic trees and interpretation of the evolutionary scenario by these authors [41]. Skerratt *et al.*[40] analysed their data using the maximum parsimony criterion in order to reconstruct the phylogeny of the haplotypes, when a more appropriate procedure would have been to use the parsimony-splits method of Bandelt and Dress [42]. Therefore, the interpretation made by Skerratt *et al.*[40] was in fact incorrect [41].

This case highlights two facts related to genetics-based studies: (a) the importance of the correct selection of genetic markers and models for analysis and (b) the possibility of genetic data (sequences) reuse and additional analysis by other research groups if uploaded to appropriate open-access databases.

Walton *et al*. [30,43] used multi-locus genotyping applied to microsatellite markers to conclude that gene flow between scabies mite populations in sympatric humans and dogs was extremely rare in northern Australia. In addition, genetic differences were detected in the same hosts from geographically distinct locations and even between different people in the same household. Most likely, it would have been impossible to obtain such results using classical epidemiological studies, since these would have required monitoring over long periods of time and a larger sample size. Similar observations were reported by Rasero et al. [32], where the geographical separation had real biological significance for the definition of mite sub-populations derived from different wild animals in Europe. The degree of genetic exchange that occurred between mites from different localities was apparently related to the geographical distance between them. Notwithstanding, wild host-derived mite populations were found clustered into three main groups: herbivore-, carnivore- and omnivore-derived *Sarcoptes* populations. The lack of gene flow between *Sarcoptes* populations may have improved parasitic adaptations and led to a so-called host-taxon-derived *Sarcoptes* mite found on European wild animals. Such an analysis of *Sarcoptes*-navigating webs would have been difficult to carry out using classical epidemiological studies.

Microsatellite markers were used by Alasaad *et al*. [24,25] to describe a new phenomenon of genetic structuring among *S. scabiei* at individual host skin-scale. Skin-scale structuring could be the result of local *S. scabiei* colonization dynamics, which would have influenced the non-random distribution of this ectoparasite after a single infestation or could simply have been the result of repeated infestations. Temporal monitoring of infected animals could also test these conclusions; nevertheless, this is a difficult task to perform in the wild or even in a controlled environment [44].

Studies of the epidemiology of *Sarcoptes* in a predator/prey ecosystem in Masai Mara in Kenya revealed that in cheetahs (*Acinonyx jubatus*) the infection with *S. scabiei* was associated with the balance between the total number of Thomson’s gazelles (*Eudorcas thomsonii*), the cheetah’s main prey, and the prevalence of infected individuals. Apparently, the high prevalence of mangy gazelles had a negative effect on cheetahs that was reduced when the number of healthy gazelles increased. This study concluded that cheetah infection with *Sarcoptes* had derived putatively from mangy gazelles [45].

This epidemiological study was followed up by genetics-based investigations using a set of nine microsatellite markers that concluded that *Sarcoptes* infestation in wild animals is prey-to-predator-wise and depends on the predator’s ‘favourite prey’. There was an absence of gene flow between both, the herbivore (Thomson’s gazelle and wildebeest, *Connochaetes taurinus*)-derived and between the two carnivore (lion, *Panthera leo*, and cheetah)-derived *Sarcoptes* populations in Masai Mara (Kenya). Lion- and wildebeest-derived *Sarcoptes* mite populations were similar yet different from the Thomson’s gazelle-derived *Sarcoptes* population. This could be attributed to *Sarcoptes* cross-infestation from wildebeest (the ‘favourite prey’ of the lion), but not from Thomson’s gazelle. The cheetah-derived *Sarcoptes* population had different subpopulations: one was cheetah-private, one was similar to the wildebeest- and lion-derived, and a third was similar to the Thomson’s gazelle-derived *Sarcoptes* mite population in places where both wildebeest and Thomson’s gazelle are ‘favourite prey’ items of the cheetah [33].

These two epidemiological and genetic-based studies have shown that classical epidemiology was able to detect putative parasite flow from gazelles to cheetahs whose existence was subsequently confirmed by the results of genetics-based studies. However, the first study did not detect parasite flow between wildebeests and lions, which was later detected by the molecular studies. This could be attributed, as mentioned above, to the abundance of healthy animals and data missing from the epidemiological study; on the other hand, the genetics-based study provided reliable information even with a low sample size.

In another example, epidemiological studies have described a significant relationship between trends in mange in the red fox and Iberian wolf; a one-year delay between them was found that suggests that scabietic foxes (*Vulpes vulpes*) were the most likely source of disease in wolves (*Canis lupus*) [46]. Recently, genetics-based studies in the same wolf population have concluded that the Iberian wolf-derived *Sarcoptes* populations had greater genetic diversity than other populations, including two different subpopulations: one similar to herbivore (red deer, *Cervus elaphus*, and southern chamois, *Rupicapra pyrenaica parva*)-derived *Sarcoptes* populations and another similar to carnivore (fox)-derived *Sarcoptes* mite populations. This suggests that potential prey (herbivore)-to-predator (wolf) *Sarcoptes* mite infection could take place between the studied host taxa [47] or be the result of the putative fox-to-wolf *Sarcoptes* infection.

The classical epidemiological study identified the fox-to-wolf *Sarcoptes* infection, while the genetics-based study added the putative herbivore-to-wolf *Sarcoptes* infection. Again, this could be attributed to the capacity of the molecular analysis to detect parasite-navigating webs with only low sample sizes, something that the initial epidemiological study failed to do. The described examples derived from Africa and Europe underline the complementarity of genetic approaches in the resolution of the limitations of classical epidemiological approaches. But it is worth noting that the opposite also applies since, although genetics can trace back the disease navigating-web of epizootic outbreaks, it cannot reveal all the causal factors involved in this navigation. Theoretically, mutations or cross-breeding of different varieties of *Sarcoptes* could help trace the source of epidemic outbreaks. Numerous scientists have studied the genetic relationships in *Sarcoptes,* but clear evidence of genetic determinism in navigating webs are still lacking. Host-parasite systems depend on ecological and epigenetic factors, and the classical epidemiological approach is very good at unraveling these relationships. For instance, scabies infection is prone to occur in individuals with identifiable immunosuppressive factors [48] and is able *per se* to induce immune-modulations in some individuals [12]. This could represent an open door for a navigating web between different host species. The cross-infectivity of *Sarcoptes* between hosts is sometimes possible [49-51] and the unraveling of the combinations of factors that can favour navigating webs is a major challenge for further mange studies. Causal factors are a key component of this question, and classical epidemiological approaches are also necessary to compensate for the limitations of genetics-based studies.

To date, classical studies still present as a potential tool to reveal pivotal epidemiological patterns; such as the effect of sex, race, age classes and socioeconomic status, together with the temporal and cyclical variation. Nonetheless, in humans no consistent epidemiological patterns are reported, and therefore, a global meta-analysis is recommended as proposed by *Sarcoptes* World Molecular Network, and the International Alliance for the Control of Scabies [5,52].

Another challenge for the classical studies is the correct diagnosis of *Sarcoptes* infections [53]. There is a lack of sensitive and reliable diagnostic tests especially for humans [54]. Scabies diagnosis is achieved via clinical signs and microscopic examination of skin scrapings. Notwithstanding, studies have shown that the sensitivity of these traditional tests is less than 50% [55]. Visible lesions are often obscured by eczema or impetigo, or are atypical, and this makes scabies diagnosis difficult. Definitive diagnosis is based on the identification of different developing stages of *Sarcoptes*, including eggs, eggshell fragments, or mite faecal pellets. Antibody detection is another alternative, but still not an effective tool. A study investigating cross-reacting IgG antibodies to the fox mite antigen in human scabies reported a sensitivity of only 48%, compared to 84% in dog mange and 80% in pig mange, which is not surprising since *S. scabiei* from humans and animals are genetically distinct [55,56]. PCR-based techniques have recently been proposed as an alternative tool for the detection of *S. scabiei* DNA in skin scrapings, but such methods are still not widely tested [57].

## Conclusions

Scabies is a paradigmatic example of how we can improve our knowledge of disease-navigating webs. Classical epidemiological studies should be integrated into genetic investigations (EG-approach) using, for instance, the statistical analysis of disease prevalence and population genetic studies of the pathological agent. Both types of study groups should identify a common platform to carry out integrated research for the sake of improving our understanding of this disease-NW, which is a cornerstone of public health.

## Competing interests

The authors declare that they have no competing interests.

## Authors’ contributions

SA, LR and XQZ conceived and designed the review, and critically revised the manuscript. SA drafted the manuscript. MS, JH, DM and RCS contributed to drafting the manuscript. All authors read and approved the final manuscript.
